# Conspicuousness and recurrence related factors of ultrasound-guided microwave ablation in the treatment of benign thyroid nodules

**DOI:** 10.1186/s12893-021-01312-1

**Published:** 2021-08-03

**Authors:** Baoying Xia, Boyang Yu, Xiaofei Wang, Yu Ma, Feng Liu, Yanping Gong, Xiuhe Zou, Jianyong Lei, Anping Su, Tao Wei, Jingqiang Zhu, Qiang Lu, Zhihui Li

**Affiliations:** 1 Department of Thyroid, Parathyroid Surgery Center, West China Hospital, Sichuan University, Chengdu, 610041 China; 2 Department of Ultrasound, West China Hospital, Sichuan University, Chengdu, 610041 China

**Keywords:** Thyroid nodule, Microwave ablation, Volume reduction ratio, Initial volume, Recurrence

## Abstract

**Objective:**

Microwave ablation (MWA) is a minimally invasive technique for the treatment of benign thyroid nodules. The purpose of this study was to evaluate efficacy and safety of ultrasound-guided MWA in the treatment of benign thyroid nodules, and to find out the recurrence related factors, so as to provide reference for future clinical work.

**Methods:**

This study retrospectively analyzed the patients who received ultrasound-guided MWA for benign thyroid nodules in our hospital from October 2018 to March 2020. A total of 214 patients were included in the study. We assessed thyroid volume changes (represented by volume reduction ratio VRR), the energy per 1 mL reduction in nodular volume (represented by energy volume ratio ΔE), the serum levels of free triiodide thyroid hormone (FT3), free thyroxine (FT4), thyrotropin (TSH) and complications after MWA treatment.

**Results:**

There were a total of 306 nodules in 214 patients, including 183 (85.51%) females and 31 (14.49%) males. The median diameter and volume of the nodule were 33 mm and 8.01 mL. The VRR at 1 month, 3 months, 6 months and 12 months were 40.79%, 60.37%, 74.59% and 85.60%, respectively. In addition, MWA had a better ablation effect for small nodules (initial volume ≤ 10 mL). In recurrent studies, we found that ΔE was an independent risk factor for benign thyroid nodules (P < 0.05).

**Conclusions:**

Ultrasound-guided MWA is effective and safe in the treatment of benign thyroid nodules. In addition, it has little damage to surrounding tissues and no effect on thyroid function. Especially, the nodules with smaller initial volume, the treatment is better. On the other hand, the energy per 1 mL reduction ΔE in nodular volume may be associated with nodular recurrence, which requires further follow-up for longer periods. At this stage, we consider that ultrasound-guided MWA can be used as one of the main clinical treatment methods for benign thyroid nodules.

## Introduction

Thyroid nodules are very common in the population and account for 65–95% of all thyroid diseases [1–3]. Thyroid nodules are often detected by color Doppler ultrasound. More than 90% of thyroid nodules are benign [4]. Current guidelines indicate that some asymptomatic thyroid nodules may be continued to be observed, and that further treatment is often required when symptomatic compression or cosmetic effects occur. Initially, benign thyroid nodules can be treated with open surgery and levothyroxine therapy [5]. On the one hand, the efficacy of levothyroxine is not significant [6–8]; On the other hand, the operation was performed under general anesthesia and was evaluated by CEUS, which left unknown sequelae for the patient. In addition, the operation inevitably causes trauma and surgical scar [9, 10]. Later, thermal ablation was applied to treat benign thyroid nodules in clinical practice due to its traceless and significant efficacy.

Because I-131 is radioactive, it has some effect on reducing the size of small nodules. Some studies [11, 12] confirmed the effectiveness of the combination of microwave ablation (MWA) with radioiodine therapy (RIT). The combined therapy is an innovative and conservative approach, which can reduce I-131 dosage and length of hospital stay, and which would become a safe alternative to surgery for the treatment of very large benign nodular goiters. In another study [13], where MWA and RIT treated thyroid nodules separately, 20% hypothyroidism occurred in RIT, while was not reported in MWA. In this regard, MWA may be a great alternative to RAI due to its advantages in terms of non-exposure to radiation and lower risk of hypothyroidism after treatment. In addition, I-131 has been reported to induce radiation thyroiditis and elevated TRAb level [14]. So I-131 dose should be used accurately, too much or too little will affect the efficacy. In addition, RIT should only be used for patients with normal iodine uptake, and it is not recommended for pregnant women. Currently, the ablation treatment of benign thyroid nodules has achieved a relatively significant effect in clinical practice. Whether the combined dose of I131 can be gradually reduced still needs more research data to support.

Thermal ablation includes laser ablation (LA), radiofrequency ablation (RFA), high-intensity focused ultrasound (HIFU) and microwave ablation (MWA), among which RFA is the most common [15]. RFA has been routinely used for the treatment of liver, vascular and other diseases [16]. In addition, RFA is also effective in the treatment of benign thyroid nodules, but it has been reported that it is less effective in the treatment of solid nodules [17]. HIFU is completely non-invasive because the principle of HIFU is to destroy the nodules through ultrasonic heat generation. In the study of Trimboli, Lang et al. [18, 19], the 1 year VRR of HIFU was 40% ~ 62%, which was slightly lower than other thermal ablations. Therefore, HIFU is not very effective in the treatment of large nodule. In addition, HIFU is often not the first choice for thermal ablation because of the high cost of treatment. As a minimally invasive technique, MWA has been gradually developed in clinic due to its great effect in the treatment of benign thyroid nodules. Therefore, this study retrospectively analyzed the volume reduction ratio (VRR) and energy volume ratio (ΔE) of thyroid nodules after ultrasound-guided MWA to explore the therapeutic effect, safety and risk factors for thyroid nodules.

## Materials and methods

### Clinical data

The study selected patients who underwent ultrasound guided MWA of benign thyroid nodules at West China Hospital of Sichuan University from October 2018 to March 2020. A total of 214 patients with benign thyroid nodule treated by MWA were included in this study, including 31 males and 183 females. The inclusion criteria were: (1) The benign thyroid nodules were confirmed by ultrasound guided or fine needle aspiration; (2) have contraindications to surgery such as complaints of pressure symptoms, throat constraint, and/or swallowing difficulty; (3) esthetic problems; (4) refuse radioiodine therapy or surgery; (5) with normal thyroid function. The exclusion criteria were: (1) Biopsy pathology indicated malignancy or equivocal pathological results; (2) Suspicious Ultrasound malignant features (such as irregular margins, obvious hypoechogenicity or microcalcification); (3) critical adjacency to structures such as vessels, trachea, esophagus and nerves; (4) With other thyroid diseases, such as retrosternal goiter, Graves’ disease; (5) Complicated with other serious diseases: acute infection, severe heart and lung disease, coagulation dysfunction;(6) Previous radiotherapy for the head and neck; (7) incomplete clinical data.

### Equipment and instruments

*MWA equipment* The microwave instrument (KU-2000 Kangyou Medical, Nanjing, China) including a microwave generator, a flexible low-loss coaxial cable and a cooled shaft antenna were used to deliver microwave energy. The generator is capable of producing 1–100 W of power at 2450 MHz. The internally cooled needle microwave antenna is 16-gauge, and coated with polytetrafluoroethylene to prevent adhesion. It is 1.6 mm in diameter and 3 mm in length.

### Treatment

(1) The patient was placed in a supine position, and the thyroid nodule was located by ultrasound; (2) Local anesthesia of the affected surface skin was performed with 2% lidocaine; (3) Normal saline is injected around the affected nodule to form a barrier to protect the surrounding tissues and nerves; (4) Ablation needles were inserted into the affected nodules under ultrasound guidance. The nodules were performed by multi-point and multi-layer ablation until the nodule was covered by a strong echo generated by thermal energy; (5) For cystic nodules containing fluid, cystic fluid should be withdrawn before MWA; (6) After this, ultrasound was used to check whether the nodule ablation was complete and to confirm no active bleeding; (7) Finally, the puncture site is disinfected and covered with a sterile dressing.

### Data collection

Collect basic information of patients, including name, admission number, gender, age, etc. Preoperative benign thyroid nodules, size, nature, duration of ablation, ablation power, complications, average length of stay. The size of benign thyroid nodules and the levels of TSH, FT3 and FT4 in plasma at 1, 3, 6 and 12 months after the operation.

### Evaluation and follow-up

The nodules were divided into three types: cystic nodules (liquid composition > 90%); Solid nodules (liquid composition ≤ 10%); the rest were cystic and solid mixture nodules. Small nodules were defined as initial volume ≤ 10 mL [20].

The therapeutic effect after treatment was calculated via ultrasound follow-up at 1, 3, 6 and 12 months, and the nodule volume (V) and lesion volume reduction ratio (VRR) were calculated: V = length (cm) × width (cm) × height (cm) × 0.524 (mL). Volume reduction ratio (VRR), that is the percentage of the reduction in the nodule’s volume during following up compared to the initial volume: VRR = (V_0_ − V_x_)/V0 × 100%. The VRR of benign thyroid nodules > 50% in half a year was considered effective [21]. The recurrence was defined as an increase of more than 50% in the volume of the nodule in the total ablation area compared to the previous ultrasound examination. The energy consumed for each 1 mL of nodular volume reduction can be calculated as follows: ΔE = PT/(V_0_ − V_x_). (P: ablation power; T: ablation time) Hematological indicators, such as TSH, FT3, and FT4, were detected to assess thyroid function before and after ablation. Postoperative complications such as hoarseness, hypocalcemia, skin lesions, and hematoma were used to evaluate the safety of MWA.

### Statistical analysis

We used statistical software SPSS 23.0 to analyze the data. The Kruskal–Wallis rank sum test was used to analyze the significance difference of skewed distribution data. Chi-square test was used to compare categorical variables. Multivariate analysis of microwave ablation effect was performed using binary Logistic regression model. The results are presented as odds ratios (ORs) with a 95% confidence interval (CI). The difference is statistically significant when *P* value is less than 0.05. Furthermore, we used Origin 9.0 to draw the statistical graph.

The informed consent was obtained from participants and legal guardian of participants below 18 years of age. This retrospective study was approved by Ethics Committee on Biomedical Research, West China Hospital of Sichuan University (approval number: 2020599). All methods are carried out in accordance with the relevant guidelines and regulations (The Declaration of Helsinki).

## Results

### General information

The median age was 45.84 ± 14.32 (11–91) years. The mean ablation time of patients treated with MWA was 7 min 25 s, and the mean ablation power was 30.52 (30–40) W. The mean total hospital stay for ablation was 2 (1–7) days. The median follow-up time in this study was 10.12 ± 2.78 months. There were 148 cases of single nodules and 66 cases of multiple nodules.

### Benign thyroid nodule characteristics

In this study, 306 thyroid nodules were ablated by microwave in 214 patients. The nodule had a median maximum diameter of 33 mm and a mean volume of 8.01 mL. The number of nodules in the lower thyroid lobe was the largest, accounting for 37.58% (115/306). The number of cystic nodules, solid nodules and mixed nodules was 26 (8.50%), 78 (25.49%) and 202 (66.01%), respectively. The volume of nodules (≤ 10 mL) before MWA was dominated by small nodules, accounting for 58.17% (178/306) (Table 1).

**Table 1:** Profiles of thyroid nodules treated with MWA

Characteristic	All nodules (N = 306)	Percentage (%)
Largest diameter (mm)	33 (4–70)	
Volume (ml)	8.01 (2.25–16.98)	
Location		
Upper lobe	94	30.72
Middle lobe	97	31.70
Lower lobe	115	37.58
Solidity		
Cystic	26	8.50
Solid	78	25.49
Mixed	202	66.01
Volume (ml)		
v ≤ 10	178	58.17
v > 10	128	41.83

### Efficacy evaluation

**Fig. 1:**
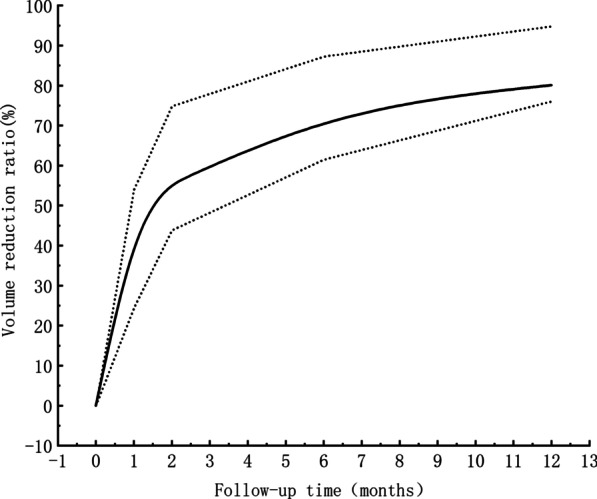
Volume reduction ratio of benign thyroid nodules during the follow-up time. The black line represents the median, and the shaded area represents the discrete variable trend of the volume reduction ratio during follow-up

**Fig. 2:**
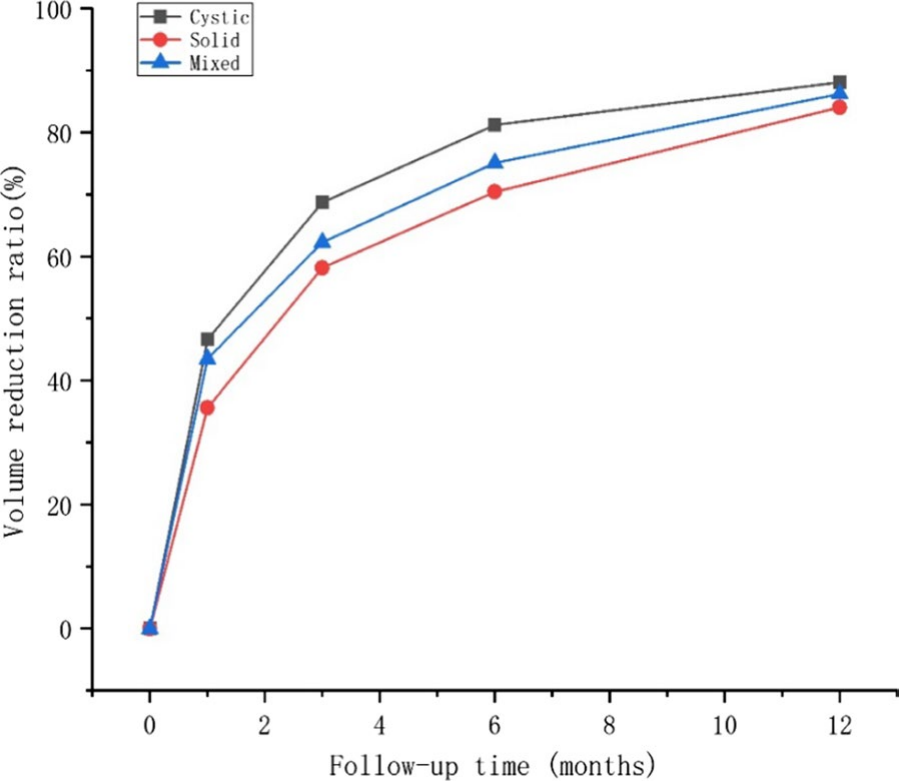
Cystic nodule, solid nodule, and mixed nodule changed in volume reduction ratio (VRR) with the follow-up time

**Fig. 3:**
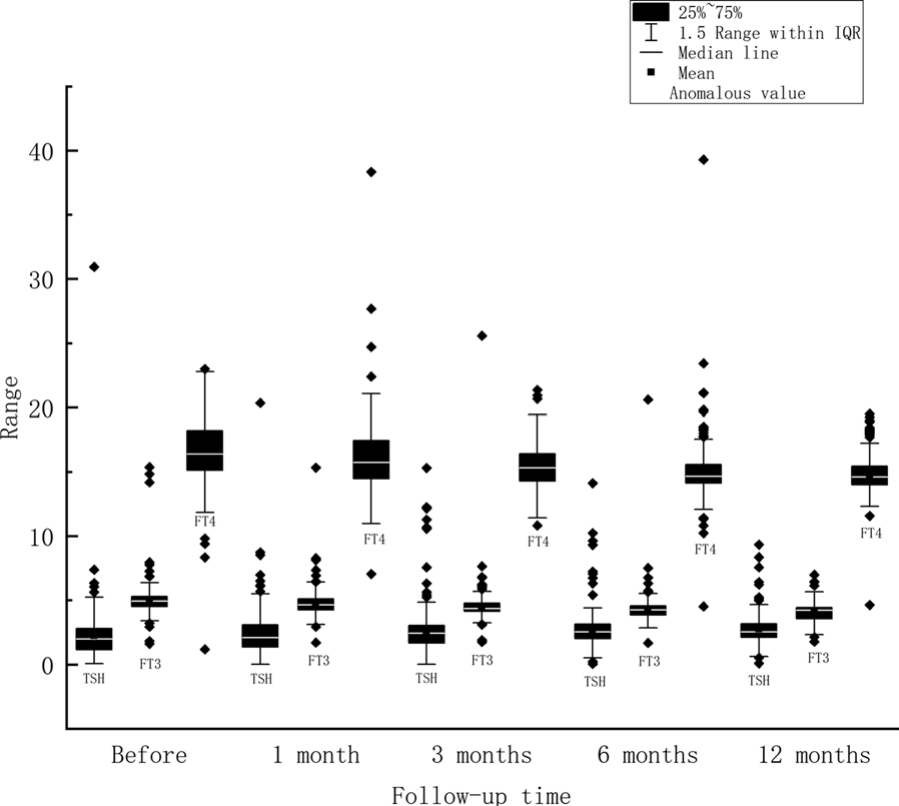
Plasma levels of TSH, FT3 and FT4 in patients with benign thyroid nodules during follow-up at baseline, 1, 3, 6, and 12 months

After MWA treatment, the VRR of benign thyroid nodules increased over time (Fig. 1). The annual overall average VRR reached 85.60%. In our study, we found that ultrasound-guided MWA has a better therapeutic effect on cystic nodules. However, there was no significant difference in VRR between different thyroid nodule types (cystic, solid, mixed) 6 months after MWA (P > 0.05) (Table 2, Fig. 2). In addition, we found that the mean hormone levels of thyroid function indexes (TSH, FT3, FT4) after ablation were all within the normal range. Moreover, the levels of FT3 and FT4 were not significantly different before and after ablation, while the levels of TSH were statistically different (Table 3, Fig. 3). In univariate analysis, nodule initial volume was found to be statistically significant with VRR (Table 4). In binary Logistic regression analysis, we found that the initial volume of thyroid nodules was an independent risk factor for the treatment of benign thyroid nodules by MWA (P < 0.05) (Table 5).

**Table 2:** VRR of different nodule types

	1 month	3 months	6 months	12 months
Total	40.79	60.37	74.59	85.60
Solidity				
Cystic	46.64	68.72	81.21	88.12
Solid	35.59	58.19	70.46	84.08
Mixed	43.50	62.28	75.11	86.24
*P* value	0.001	0.008	0.140	0.389

**Table 3:** Thyroid hormone changes during follow-up period

	Before	1 month	3 months	6 months	12 months	*P* value
TSH (mU/L)	2.24 ± 2.34	2.43 ± 1.94	2.65 ± 1.96	2.72 ± 1.54	2.78 ± 1.31	0.026
FT3 (pmol/L)	5.07 ± 1.40	4.77 ± 1.07	4.51 ± 1.62	4.32 ± 1.32	4.13 ± 0.73	0.445
FT4 (pmol/L)	17.09 ± 5.52	16.09 ± 2.71	15.64 ± 2.91	15.08 ± 2.44	14.83 ± 1.65	0.447

**Table 4:** Single factor analysis of MWA ablation effect

Characteristics	VRR > 50%	VRR ≤ 50%	*X*^*2*^	*P*
Gender				
Male	40	4	1.668	0.197
Female	214	48		
Age				
≤ 60	210	44	0.115	0.735
> 60	44	8		
Location				
Upper	81	13	1.421	0.491
Middle	81	16		
Lower	92	23		
Solidity				
Cyst	20	6	0.944	0.624
Solid	62	16		
Mixed	168	34		
Initial volume				
≤ 10 mL	141	37	4.340	0.037
> 10 mL	113	15

**Table 5:** Logistic regression analysis of MWA ablation effect

Variable	B	β	Wald	P	OR	95%CI
Initial volume (≤ 10 mL, > 10 ml)	− 0.681	0.331	4.236	0.040	0.506	(0.264, 0.968)
Constant	2.019	0.275	53.998	0.000	7.533	

### Recurrence correlation assessment

Among 306 benign thyroid nodules, 7 nodules recurred, accounting for 2.29%. The maximum diameter, initial volume and ablation power of nodules were significantly higher in the recurrence group than in the non-recurrence group. In addition, the recurrence group had a higher energy to volume ratio than the non-recurrence group, which was statistically different (P < 0.05). In other words, the more energy required to reduce the volume of 1 mL of nodules, the more likely it was to be associated with recurrence (Table 6).

**Table 6:** Factors related to recurrence of benign thyroid nodules

	Recurrence group	Non-recurrence group	*X*^***2***^	*p* values
Number of patients	7	299	–	–
Maximum diameter of nodule (mm)	46 (38.5–48.5)	33 (20–42.5)	0.515	0.473
Initial volume (mL)	13.86 (6.06–15.42)	7.86 (2.25–17.04)	0.192	0.661
ablation power (w)	31.10 ± 2.62	30.53 ± 1.84	0.370	0.543
Energy volume ratio ΔE (kJ/mL)	2.66 (2.03–5.85)	0.94 (0.48–1.81)	8.124	0.004

### Safety assessment

Among 214 patients treated with MWA, none had serious complications. However, one patient developed intracapsular hemorrhage and recovered within a month. Another case developed recurrent laryngeal nerve injury but recovered within 2 months.

## Discussion

In this study, we retrospectively analyzed 214 patients who underwent ultrasound—guided MWA. After 10.12 ± 2.78 months of follow-up, the VRR of benign thyroid nodules was up to 85.60%. Univariate analysis showed that there was a statistical difference between the initial volume of nodules and the effect of MWA. Logistic regression analysis showed that MWA had a good effect on the initial volume of nodules less than or equal to 10 mL. Furthermore, the serum levels of FT3, FT4 and TSH were all within the normal range, indicating that MWA would not impair thyroid function.

The therapeutic effect of MWA has been proved clinically at home and abroad. It has a good therapeutic effect on benign thyroid nodules. Numerous studies have shown that VRR can reach 24.0–51%, 54.8–75.1%, 68.7–85.2%, 75.8–96.4% respectively after 1, 3, 6 and 12 months by MWA [1, 5, 22–25]. Our study showed similar outcomes. Last but not least, the goal of ablation is not complete removal of the nodule, but to achieve a stable clinical result with the minimum possible rate of complications.

Our results are consistent with those reported by Liu, Yue, Wu et al. [17, 24, 26], none of the patients treated with MWA had serious complications. Only one case of intrathyroid hemorrhage and postoperative hoarseness occurred in our study, but all recovered within 2 months. Hu et al. [23] found that 2.3% of the sound changes after MWA, but all recovered within two weeks. In conclusion, the treatment of benign thyroid nodules by MWA is characterized by fewer complications and easy recovery. In our hospital, all patients were discharged one day after MWA, and long-term follow-up was conducted to track their recovery. Some patients with throat discomfort are generally advised to drink more water and appropriate atomization. And for patients who develop hoarseness and do not get any relief within a month we do a laryngoscopy. At the same time, it is necessary to recheck haematological indicators such as PTH and calcium every time. If hypocalcemia is found, appropriate calcium supplementation is recommended.

In our study, Logistic regression analysis showed that the initial volume of nodules, especially small nodules (volume ≤ 10 mL), was a risk factor for the effect of MWA. 2020 European Thyroid Association Clinical Practice Guideline for the Use of Image-Guided Ablation in Benign Thyroid Nodules [20] state that the smaller the treated nodule, the higher the volume reduction. Cesareo et al. [27] obtained similar results in their study, suggesting that ablation can effectively reduce benign thyroid nodules, especially small ones. Lee et al. [21] found that ablation was more effective in the treatment of small nodules with a volume less than 4 mL (P = 0.030).

Heck et al. [28] proposed that the serum levels of T3, T4, TSH, thyroglobulin (Tg), anti-TG, thyrotrophin receptor (TRAb), and thyroid peroxidase (anti-TPO) showed no significant changes after the MWA and the follow-up of half a year. Erturk MS et al. [29] pointed out that the effect of MWA on thyroid function had no significant difference at 6 months, but the effect was significant at 24 h. In our study, we reached the same conclusion that the serum levels of thyroid function index were all within the normal level after MWA and decreased compared with that before ablation. Therefore, we have reason to believe that MWA has no effect on thyroid function.

In the study on risk factors related to recurrence, we found that the maximum diameter, initial volume and ablation power of nodules in the recurrence group were larger than those in the non-recurrence group. The energy per 1 mL reduction in nodular volume was greater in the recurrence group than in the non-recurrence group, which was a significant difference. However, Wang et al. [30] indicated that initial volume, vascularity, and the energy per 1 mL reduction in nodular volume were all risk factors for recurrence of benign thyroid nodules treated with ultrasound guided microwave ablation. In addition, Korkusuz et al. [31] revealed that solid nodules require more the energy per 1 mL reduction than cystic and mixed nodules. Therefore, for large volume nodules requiring high power and long time ablation in clinical practice, they are often prone to recurrence.

The comparison between MWA and open surgery has been thoroughly studied. It is generally believed that MWA is less invasive than surgery, with faster recovery and no scarring. The European guidelines [20], Korean guidelines [32], American guidelines [33] and Chinese guidelines [34] all indicated that thermal ablation is currently an innovative and alternative method for the treatment of benign thyroid nodules. Among them, RFA and LA have been proved to have satisfactory clinical results by a large number of literatures. Treatment options for MWA and HIFU are still being evaluated. Liu, Sy et al. [35] pointed out that stress response of ultrasound-guided MWA to treat with nodules was significantly lesser than that of surgery. Postoperative IL-6, IL-8 and TNF in MWA group were lower than those in surgery group (P < 0.05). RFA is one of the most widely studied types of thermal ablation. RFA has been shown to be effective in reducing benign thyroid nodules in numerous studies. Trimboli et al. [36] showed that the energy delivered with RFA is the only technical parameter significantly correlated with the VRR of thyroid nodules. In contrast, the energy volume ratio (ΔE) proposed in our study also can predict recurrence risk factors greatly. In the study, the energy parameter set by MWA was 30 W. In some cases, the ablation power may be increased in the presence of large nodule. LA is also a common thermal ablation technique, but the ablation effect is not as good as RFA. Trimboli et al. [37] showed that it took 3 years for LA to stabilize the size of the nodule after ablation, while only 2 years for RFA. In addition, because of the high cost of LA, its clinical application is less common than that of RFA and MWA.

This study still has some limitations. First, this was a retrospective study, with no control group. Second, the follow-up time in this study was not long enough so that the VRR reached a plateau value. Third, symptom scores and cosmetic scores were not used for quantitative evaluation. Therefore, long-term, quantitative and prospective studies are needed to further verify the conclusions in the future.

## Conclusion

In summary, our study shows that ultrasound-guided MWA is effective and safe in the treatment of benign thyroid nodules. In addition, MWA has a good effect on different types of thyroid nodules (cystic, solid and mixed). Especially for the initial small volume of nodules, treatment is better. MWA is characterized by better protection of thyroid function, fewer complications, superior esthetic results and a lower recurrence rate. The energy per 1 mL reduction in nodular volume was also associated with recurrence. In clinical practice, we believe that ultrasound-guided MWA is also an alternative treatment for benign thyroid nodules.

## Data Availability

The datasets used and analyzed during the current study are available from the corresponding author on reasonable request.
